# “When a Father feels Excluded”: A Qualitative Study Exploring the Role of Fathers in the Women, Infants, and Children (WIC) Supplemental Nutrition Program

**DOI:** 10.1080/17482631.2021.1932026

**Published:** 2021-06-22

**Authors:** Dan K. Dychtwald, Girija Kaimal, Linda M. Kilby, Cynthia Klobodu, Brandy-Joe Milliron

**Affiliations:** a Department of Nutrition Sciences, College of Nursing and Health Professions, Drexel University, Philadelphia, PA, USA; b Department of Creative Arts Therapies, College of Nursing and Health Professions, Drexel University, Philadelphia, PA, USA; c NORTH, Inc., Philadelphia, PA, USA

**Keywords:** Fathers, WIC, paternal involvement, birthing, breastfeeding, infant health, preterm birth, low birthweight

## Abstract

**Background**: Evidence suggests that men can play a key role in influencing maternal health behaviours, potentially affecting birthing outcomes. However, that role may not be fostered in safety net programmes like the Special Supplemental Nutrition programme for Women, Infants, and Children (WIC), a programme for which men do not qualify.

**Purpose**: The primary objective of this research was to explore the experiences, expectations, and attitudes of men towards WIC.

**Methods**: This qualitative study employed semi-structured interviews of couples recruited at Philadelphia WIC. Data were analysed using thematic analysis.

**Results**: Eight couples completed the interviews (16 independent interviews). Among participating fathers, only two fully participated in WIC. Barriers to participation was the primary theme identified as participants shared challenges from multiple sources. Subthemes, including fears of coercion, masculinity, and the unacknowledged role of fathers illustrated that these barriers were both internal and external to WIC and in alignment with the framework of the social ecological model (SEM).

**Conclusion**: These findings indicate that paternal involvement is limited due to numerous barriers, including those attributable to WIC. Future research should investigate these barriers and their intersectionality, as well as the appropriateness of WIC as an organization to foster paternal involvement.

## Introduction

Until recently, paternal involvement (PI) has been an understudied yet critical component to improving maternal and child health (MCH) outcomes (Bond, 2010). Several studies have provided important evidence of the positive influence of paternal involvement on MCH. This includes reducing preterm (gestation < 37 weeks) and low birthweight (LBW) births (birthweight < 1500 g) (CDC, 2020a) and increasing breastfeeding initiation and continuation (Mitchell-Box and Braun, 2013; Martin et al., 2007; Redshaw & Henderson, 2013). Federally subsidized nutrition programmes, such as the Special Supplemental Nutrition programme for Women, Infants, and Children (WIC), are also tasked with improving MCH. WIC is a safety-net programme, funded nationally but administered by both the USA Department of Agriculture’s (USDA) Food and Nutrition Service (FNS) and at the state level. As of 2020, WIC served an average of 6.2 million infants, children, pregnant, and post-partum women per month (Kline et al., 2020). The programme, whose mission is to “safeguard the health of low-income women, infants, and children up to age 5 who are at nutrition risk by providing nutritious foods to supplement diets, information on healthy eating, and referrals to health care” (USA Department of Agriculture, 2018) provides medical screenings, breastfeeding support, nutritional counselling and education, and electronic benefits transfer (EBT) cards for the procurement of nutritious food. As the name implies, men are ineligible for WIC benefits, but their partners or children may be enrolled in the programme. However, some states and local WIC offices make an effort to both welcome and invite men to be a part of the programmeme in support of their families, as well as to increase their own knowledge of nutrition (California WIC Association, n.d.). However, as WIC does not provide direct support to men, FNS does not keep nor provide data regarding their participation in the programmeme.

While both paternal involvement and programmes like WIC may play critical roles in MCH outcomes, there is no current research looking at the intersection of the two. This includes the inclusion of men in WIC programmeming and their respective experiences within the programme. The same can be said of other USDA safety-net programmes like the Supplemental Nutrition Assistance programme. According to the 2010 US Census approximately 37% of male-householder families received some form welfare/government/social service support as compared to over 58% among female-householder families (Irving & Loveless, 2015). This may explain the scant research on the receipt of welfare by these male-householder families and particularly with experienced or perceived barriers. However, a handful of studies have investigated biases expressed by those working in social services regarding a man’s need or eligibility for such government aid (Baum, 2016; Cameron et al., 2012).

Currently, the USA’ (US) infant mortality rate is 5.3 per 1000 live births, the highest rate among the most industrialized G7 nations and comparable to countries like Gibraltar, Latvia, and Serbia (CIA World Factbook, 2020). Leading causes include disorders related to short gestation and low birthweight (Centers for Disease Control and Prevention, 2020a). Presently, US rates of preterm and LBW births have declined to 9.63% and 8.07%, respectively, with higher rates reported by non-Hispanic Blacks (Centers for Disease Control and Prevention, 2018).

The Philadelphia WIC programmeme reports a preterm birth rate of 11.89% and LBW rate of 11.97% (Khanuja, 2017), higher than both the current county averages of 10.8% and 10.4%, respectively, and the national averages (Pennsylvania Department of Health, 2019). Studies have found that the preterm birth and LBW rates may be generalizable to an interplay of socioeconomic factors and race (American Public Health Association [APHA], 2006). In fact, among Hispanic and non-Hispanic Blacks where PI is reduced based on socioeconomic status these indicators are more pronounced (Alio et al., 2010).

Besides WIC and other safety net programmemes, there are other barriers to paternal involvement in maternal child health including “systemic obstacles related to employment, and a lack of confidence stemming from social stereotypes about the expected role of a father” (NICHQ, 2021). Additional obstacles may include a poor or non-existent relationship between mother and father or the mother assuming a gatekeeper role, whether intentional or implied, whose permission is seen as necessary prior to inviting a father into an interaction with a provider (Cannon et al., 2008; McBride et al., 2005). For example, a mother’s permission may be requested before inviting a father into an examination room or having them meet with a WIC nutritionist. Additionally, there are conflicting definitions as to what connotates paternal involvement, as well as how it is measured. Some studies have focused on monetary support, while other have looked at different aspects of social support. Many more look at specific tasks during pregnancy, as well as those associated with breastfeeding (Alio et al., 2010; Martin et al., 2007; Redshaw & Henderson, 2013; Surkan et al., 2017), yet there is no universally agreed upon definition or measure. PI has been shown to decrease rates of preterm and LBW births by reducing negative maternal health behaviours, including smoking, alcohol consumption and poor dietary intake. In addition, women are more likely to seek earlier antenatal care and maintain ongoing appointments (Alio et al., 2010; Martin et al., 2007). This research supports the rationale that increased PI among WIC families may result in reductions in preterm and LBW births. Beyond improved pregnancy outcomes, Cabrera et al. (2008), Cook et al. (2005), and Shannon et al. (2009) in separate studies found that the greater the PI during pregnancy, the greater the father’s involvement with the child through the life course.

Fathers, who have been referred to as “underutilized breastfeeding resources,” also influence the decision to breastfeed (Banks et al., 2013; Sweet & Darbyshire, 2009). According to *The US Breastfeeding Report Card*, nearly 85% of US babies born in 2017 were breastfed at some point during infancy. However, by 6 months of age, only about 58% were breastfed, roughly 26% exclusively. By 12 months, only 35% of infants were being breastfed in some form (Centers for Disease Control and Prevention, 2020a). A national immunization study analysing breastfeeding prevalence by race and WIC eligibility concurred between 2013 and 2014. Just over 73% WIC-enrolled mothers initiated breastfeeding, lower than the 90.5% among WIC-ineligible mothers. At 6 months, prevalence dropped by over 50% in both WIC-enrolled and WIC-ineligible infants with rates of 39.1% and 68.4%, respectively (Centers for Disease Control and Prevention, 2013). In a separate study, Redshaw and Henderson (2013) found that higher rates of PI were reported among middle and high-income families compared to low-income families.

WIC programmeming may be a missed opportunity to increase PI among participating families as paternal support could result in higher rates of breastfeeding initiation and continuation in lower income households. While at least one local WIC agency has developed male-centred programmes to foster PI, no national effort exists. That programme developed by Texas WIC used men as peer counsellors to train fathers to support breastfeeding among their partners. While the programme resulted in improvements in breastfeeding initiation, the programme was eliminated due to lack of peer counsellors (Stremler & Lovera, 2004). In several instances, child welfare service providers report being uncomfortable involving men in their interactions with families due to reports of domestic violence (Cameron et al., 2012). This may also be the case for WIC (California WIC Association, n.d.).

**Figure:**
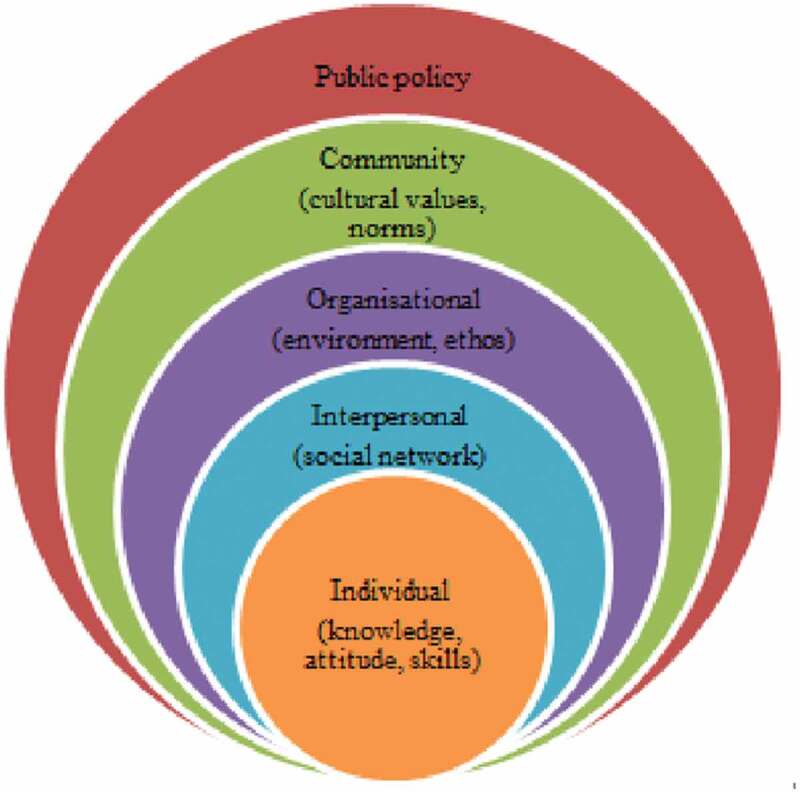

**Figure 1.**
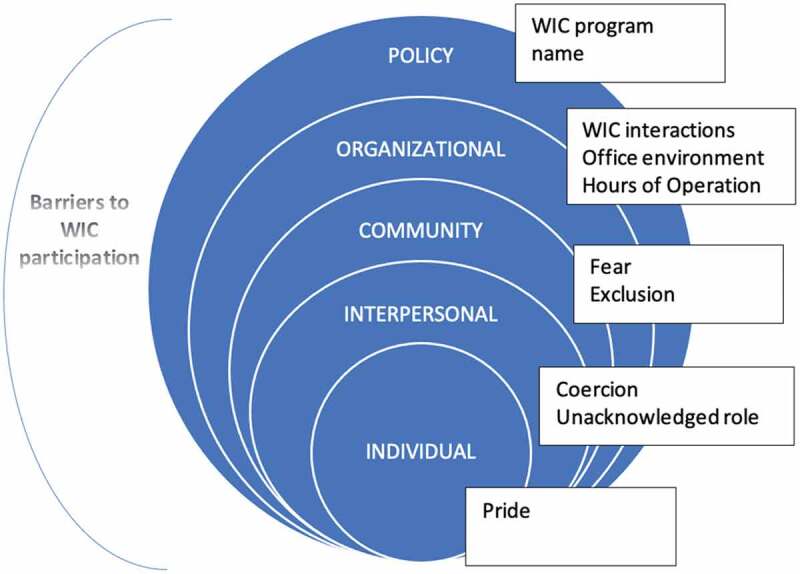
Theme and subthemes as they align with the Social Ecological Model

Questions remain, however, as to how WIC incorporates men into programmeming and if there may be missed opportunities to better leverage PI in improving MCH outcomes. This current study addresses these questions by exploring the experiences of men with WIC and their full-participation in the programme using the Social Ecological Model. Developed by sociologists in the 1970s, the social ecological model studies how behaviours are formed or influenced based on characteristics from individuals, communities, organizations, policy, and the levels in between (Borgen Project, 2017). In addition to the unique nesting structure of these independent levels, all of the level also intersect (Figure 1), indicative of the effect of their interplay. “In examining these intervals and how they interact and overlap, public health experts can develop strategies to promote wellbeing in the U.S. and abroad” (Borgen Project, 2017). In terms of full participation, for the sake of this research, it is defined and measured by the male being fully engaged throughout the interactions with WIC including being addressed directly by WIC personnel; included in nutrition education, and counselling; included in questions about the family; and able to negotiate the EBT card for the procurement of WIC-approved food. As such, the primary objective of this study is to document the experiences, expectations, and attitudes regarding WIC among men with enrolled WIC-qualifying partners or children concerning: 1) methods of recruitment and enrolment; 2) interactions with WIC personnel; 3) WIC-Ed course content; and 4) WIC administrative policies, procedures, and intentions to meet the needs of men.

## Methods

### Study design and sample

This qualitative pilot study utilized a semi-structured interview format with couples from the WIC programmes in Philadelphia. A criterion-based sampling strategy was used to select participants, focusing on only families actively participating in WIC programmes. Recruitment was conducted using flyers and in-person solicitation by the primary investigator at the eleven Metro-Philadelphia WIC offices. Participant eligibility criteria included: (1) age of 18 years or older; (2) current WIC enrolment or prior enrolment within the last 6 months; (3) existing relationship between WIC-enrolled mother and the male providing support during that enrolment period; (4) English-speaking; and (5) no impairment that may impact ability to complete the interview. Regarding this study, an existing relationship was not defined by marital status, but by the fact a pregnancy had resulted due to the interaction of the parties.

Sixteen participants composed of eight couple/dyads participated in semi-structured interviews. Each dyad was composed of one female and one male participant (male (n = 8) and female (n = 8)). Interviews were conducted at one of the 11 Metro-Philadelphia WIC offices in a private office to ensure confidentiality with each participant being interviewed individually. Prior to each interview, each participant was engaged in the written informed consent process by the primary investigator and subsequently gave consent. Each participant was then provided a brief demographic questionnaire that included age, gender, race, relationship status, education, and household income. Couples were then asked which participant wanted to be interviewed first, as the protocol did not require a specific order. At the conclusion of the interviews, couples received a 25 USD retail gift card. Interviews, each one conducted by the primary investigator, occurred between February 2016 and December 2016 and lasted between 25 minutes and 3 hours. Interviews were audio-recorded along with notes regarding study participants’ verbal and non-verbal cues. Investigator notes were consolidated in post-interview memos and used to aid in data analysis. The Institutional Review Board for Adult Social/Behavioural human subjects protection (masked for review) in Philadelphia, Pennsylvania approved the study protocol granting it an exempt status.

### Interviews

Each participant was interviewed once. The interview format was semi-structured with separate interview guides developed for men and women, each with five primary questions. The interview guide was an original construction developed after a review of the relevant literature, as no prior research had been done regarding men’s experiences with WIC. As the interview guide was an original construction of the primary investigator and the study itself was a pilot, the guide was neither piloted nor reviewed except by members of the study team.

Participants were asked about their experiences with the WIC programme from both the male and female perspective. Experiences with WIC were explored including initial contact with WIC, nutritional counselling, and breastfeeding support, along with father’s perceptions and attitudes regarding the interactions. Four of the questions focused on experiences, beliefs and attitudes regarding the father’s actual and perceived role during pregnancy and breastfeeding, and the participation of the father in WIC. A fifth question asked whether WIC was an appropriate programme in which to advocate for PI. Each of the primary questions was supplemented with follow-up questions and probing. Questions regarding PI in WIC along with pregnancy and breastfeeding were included to elucidate the differences in WIC participation as compared to conventionally accepted paternal roles. An informal interview structure was also utilized to build rapport and develop trust, allowing study participants to fully express their stories, beliefs, and views.

### Data analysis

Interviews were recorded and transcribed verbatim. Primary analysis was conducted by the primary investigator using thematic analysis to analyse the data, employing an inductive coding approach to identify and define themes (Braun & Clarke, 2006). A preliminary codebook was developed based on topic domains of WIC, pregnancy, and breastfeeding participation. Sub-codes were identified based on PI or the lack thereof. Upon completion of the codebook, all transcripts were imported into NVivo qualitative analysis software (v.11). The software was used to supplement manual analysis verifying codes, patterns, and themes, as well as identify differences by gender and participation in the various paternal roles. Query reports were generated along with word trees based off frequently cited words, phrases, ideas and concepts.

Check coding was conducted post analysis to ensure validity of the findings (Miles et al., 2019). This secondary review included discussion of the analytic approach and findings with the research team which includes the co-authors. This validity check of team debriefing (Shenton, 2004) resulted in a redefinition of primary themes finding that prior identified subthemes mirrored the constructs of the Social Ecological Model (SEM), “a theory-based framework for understanding the multifaceted and interactive effects of personal and environmental factors that determine behaviours, and for identifying behavioural and organizational leverage points and intermediaries for health promotion within organizations” (UNICEF, 2012). The framework—individual, interpersonal, community, organizational, and policy allowed for the exploration of each level, as well as the interplay between levels.

## Results

The study sample included 8 couples/dyads composed of one male and one female apiece, with all female participants self-identifying as WIC recipients. Participants ranged in age from 24 to 38 years with 13 self-identifying as African American and 3 identifying as White of European descent. All the couples were married. The average number of children was 2 and 3 for females and males, respectively. The mean number of people in the household was 4 for each group with females reporting 1 person receiving WIC support in their household and males reporting 2 people receiving WIC support in their household. Four out of 8 females had a college or advanced degree compared to 3 out of 8 males. Three females reported a household income

above 40,000 USD whereas 6 males reported a household income above 40,000. USD An overview of participant demographics by gender for the sample is shown in Table I.

**Table I.** Participant demographics by gender

Characteristic	Female (n = 8)	Male (n = 8)
Age range, years	24–37	26–38
Mean age, years	31.6	32.9
Race		
Black	6	7
White of European descent	2	1
Married	4	4
Mean number of children	2	3
Mean number in household (HH)	4	4
Mean number in household receiving WIC	1	2
Level of education completed		
High School	2	2
Technical School	2	3
College	3	2
Advanced Degree	1	1
Household Income		
0–10,000 USD	1	2
10,001–20,000 USD	1	0
20,001–30,000 USD	1	0
30,001–40,000 USD	2	0
40,001 USD+	3	6

The analysis focused on identifying themes related to the experiences, perceptions, and beliefs of men regarding WIC from the male and female perspective. One primary theme and nine subthemes were identified. The nine subthemes corresponded to the levels of the SEM (Figure 1). The primary theme, barriers to participation, encompassed the challenges men face as they try to fully participate in the WIC programme. Full participation related to the active engagement of the father throughout the WIC interaction, including certification, nutrition counselling, and working with the breastfeeding peer counsellor. Of the eight men interviewed, only two men were found to be fully participating in WIC. Both were over the age of 35 and college educated. An additional two males were categorized as WIC attendees, but as neither left the waiting area, they were not found to meet the definition of full participation. The nine subthemes included pride, fear, fear of coercion, unacknowledged roles, feelings of exclusion, hours of operation, WIC programme interactions, WIC office environment, and WIC name. Findings are presented by SEM level and subtheme (Supplementary Table).

### Individual

#### Pride of masculinity

The subtheme of pride, as discerned from the interviews, related to threats to personal pride, self-esteem, or personal privacy. It was a theme not brought up by participating men, but instead by their partners, which may be indicative that these fathers found even sharing concerns regarding masculinity as threat to that pride. In addition, it was a barrier identified only by female respondents self-identifying as African American. These women shared specific concerns regarding male pride, in particular, associating WIC with welfare. This was illustrated in a variety of ways, but most often connected to the negative connotations of welfare held by men. The women shared that these negative associations with welfare sometimes forced women in the African American community to keep their WIC enrolment hidden from partners. One mother shared,

*“A lot of males think WIC is welfare … some people think welfare is bad” (African American mother of 3, age 33)*

In addition, women shared that men did not like to answer personal questions, such as those related to family structure, income, employment, and marital status. They indicated that their partners did not like to share this information in front of others. There were concerns regarding privacy and having personal information overheard or known by others. Shared one mother,

*“It’s a pride thing for men … don’t like to discuss things in front of others … privacy very important” (African American mother of 3, age 31)*

In all cases where pride was acknowledged as a barrier by the female participant, there was no paternal involvement in WIC among their participating male partner/child’s father.

### Interpersonal

#### Unacknowledged male parental role

Like pride, only women articulated the unacknowledged parental role. It was related to the male’s role within the family structure and how neither that role nor the man’s contribution was acknowledged. Shared one mother,

*“When you say women, infants, and children, I think they’re missing the … the key factor which is the mom and dad” (African American mother of 3, age 31)*

However, this barrier was not associated solely with WIC as mothers insinuated community and societal roles. Mothers nevertheless did associate participation in WIC with stronger connections to the family and felt that WIC should take a stronger role in promoting the programme to men. Stated one mother,

*“Men feel like they don’t get credit as a parent … domino effect where the father feel touched by the programme lifts his entire family” (African American mother of 3, age 31)*

That same mother added,

*“You want to see happy families and whole families and I think this gives people a chance to … push and promote that.”*

A second woman reiterated that belief sharing,

*“Being involved in the WIC programmes … means you’re involved in his life, you’re involved, you’re around.” (African American mother of 2, age 24)*

#### Fears of coercion

Fears of coercion shared by male participants related to fears of being forced to attend WIC by either their partner or the programme itself. It also related to having a lack of personal choice in deciding whether or not to participate. This was a fear expressed only by African American men. Shared one father,

*“WIC should not play a role in increasing father involvement, it’s being forced … it must be voluntary” (African American father of 3, age 35)*

Two other fathers concurred.

*“WIC should be involved with increasing father participation as long as it’s not invasive or forced” (African American father of 5, age 37)*

*“If you say WIC should increase my role, that’s kind of being forced” (African American father of 3, age 35)*

A third father agreed but focused more on the WIC participation as an induvial choice.

*“It’s up to the man advantage and show how much he wants to be involved.” (African American father of 3, age 38)*

Women had mixed beliefs regarding coercion with one woman stating that WIC attendance should be mandatory. She shared,

*“I don’t think they need to show up for every WIC visit, but I think there should be a mandatory amount of times that the father should be there.” (African American mother of 2, age 32)*

However, not all of the were in support of making WIC mandatory with several of them suggesting WIC offer an incentive to fathers as way of encouraging attendance. Shared one mother of two,

*“Maybe offering incentives to the guys to come in, um whether it be a gift card … definitely got to get the fathers involved somehow … ” (African American, age 24)*

### Community

#### Fear

This barrier was expressed only by African American participants and relates back to the barriers of privacy within the subtext of pride. This barrier exemplified distrust among the African American community about positions of power and influence. As shared by two of the study participants, these fears were aimed directly at social service organizations and safety net programmes, particularly at those that could separate the children from the father. This included programmes like WIC, the Supplemental Nutrition Assistance programme (SNAP), Housing and Rental Assistance programmes like Section 8, and other forms of welfare. Families expressed concerns that their financial inability to support their children could be used as a means to remove the children from their home. Explained one,

*“I think a lot of minorities and low-income families are afraid to ask at the hospital because they’re scared a social worker or case worker will be invited in” (African American mother of 3, age 31)*

One father shared additional concerns about privacy saying,

“*I think there should be more, more privacy. I feel like the cubicles should be … more enclosed. There should maybe even offices, not open, open cubicles because you’re dis … discussing your child’s information” (African American father of 3, age 38)*

Although actually the fear of these services was shared only by African American participates, one White father of European descent summed up those, specifically that these families and fathers may be seen as taking advantage of the system. He shared,

*“There’s like this, this fear of … poor people taking advantage of … you know, any system that’s set up to serve poor people … ” (Father of 2, age 38)*

#### Feelings of exclusion

Exemplified by the belief that men were being excluded by WIC, this barrier was shared by men and women alike. Men shared the belief and concern that if felt excluded, they would be more likely to exclude themselves from other aspects of family and community. One father shared,

*“When a father feels excluded, he wants be less involved … if they feel these people want me around, it helps them better support the woman” (African American father of 3, age 38)*

That same father shared some his prior experiences with WIC indicating this fear may be well founded as WIC staff were not necessarily unwelcoming of his presence, but they also did not expect it. He shared,

*“I think they’re expecting to see a female, so when the male does show up, um, um … they don’t make them feel uncomfortable, but again I think they’re expecting a female to show up … ”*

As paternal involvement with the WIC programme was minimal, many of the fathers were only able to speculate about how WIC might or not might be inclusive of their role. However, among the four fathers that made it to the WIC office, only two left the waiting area and were included in the appointment.

Women concurred with the feelings of exclusion shared by their partner sharing that these men grew up without fathers and did do not know how to be a dad. By acknowledging this barrier, it was inferred that WIC could be perpetuating the cycle by not creating an environment of inclusion. Shared one mother,

*“A lot of fathers … are fathers before they’re even men, you know? And they, we grow up without fathers. We don’t really know how to care for a kid, you don’t know how to be a dad, you never had one” (African American mother of 2, age 24)*

### Organizational

#### Hours of operation

This barrier pertained to WIC’s hours of operation and was cited solely by fathers. While hours could be characterized as external barriers out of the father’s control, it was a conscious choice to attend or not making the barrier internal. Many men shared concerns regarding missing work in order to attend a WIC appointment. Shared one father,

*“Her appointments are when I’m working and I’m not going to miss work to go to a WIC appointment” (African American father of 5, age 37)*

While two other fathers shared,

*“Some people work and can’t make it” (African American father of 2, age 26)*

*“they haven’t been open on, at times that were convenient for me” (African American father of 3, age 35)*

Yet that same group acknowledged they would miss work to attend an obstetrician appointment. When asked about how involved men were during pregnancy, both men and women concurred that the men were fairly involved.

#### programme interactions

While insight on this barrier was shared by many of the female study participants, three of the four men with WIC contact provided a great deal of feedback. The barrier pertained to any interaction between fathers and WIC, including WIC staff. Both men and women, while not characterizing the staff as unwelcoming, indicated that men just aren’t expected to attend. One father suggested that the programme had not accounted for their presence characterizing the barrier as a hindrance to families as WIC was not reflective of the family structure. Shared one father of three,

*“They don’t make the male uncomfortable, but they’re expecting a female” (African American father of 3, age 38)*

A second father agreed sharing,

*“Fathers are playing an important role and that’s sometimes an ignored role” (Caucasian father of 2, age 38)*

Another, acknowledging the minimal male presence, shared that WIC should make fathers feel special.

*“Men not made to feel special … not welcoming to men” (African American father of 3, age 35)*

A third advocated for a more gender-neutral setting and approach to parenting.

*“Adopt a more gender-neutral framework for parenting” (Caucasian father of 2, age 38)*

However, there was disagreement from the women about whether men were treated differently. Some women acknowledged different treatment for men, while other women saw no difference. Shared one mother,

*“They just have the mom’s name on the card and I think if they involve the male, they need to have the guy’s name on the card” (African American mother of 2, age 32)*

#### Office environment

Office environment was defined not just by furnishings, but also graphics, posters, and brochures. This definition was expanded to include the feel and vibe of the offices shared by study participants. Both men and women voiced concerns with several women sharing that offices were unwelcoming to men and women alike, characterizing them as cold and unappealing. Men shared concerns about the minimal male presence and how that alone served as a barrier. Shared several of the fathers,

“*It’s not welcoming to both participants whether its male or female” (African American father of 3, age 38)*

*“I felt like not, not bad about it, but the programme hadn’t really accounted for dads” (Caucasian father of 2, age 38)*

Mothers concurred sharing,

*“There were like literally no men at all in the like, whole area” (Caucasian mother of 2, age 37)*

*“I find it unwelcoming as a person … like it’s not a warm environment … it feels very cold” (Caucasian mother of 2, age 37)*

### Policy

#### programme name

Both men and women shared concerns over the name, WIC, which did not account for the role of fathers or of families. Men cited that the name created a barrier and intensified feelings of exclusion. One father shared,

*“Change the name of WIC to better reflect male inclusion … just the name can make men feel uncomfortable” (African American father of 3, age 38)*

Women agreed sharing the name should be more inclusive. Shared one mother,

*“When you say women, infants, and children, I think they’re missing the … the key factor which is the mom and dad” (African American mother of 3, age 31)*

Suggestions included replacing “women” with either families (FIC) or parents (PIC).

## Discussion

Men are important to MCH. Prior research has shown the influence of PI on maternal health behaviours affecting birthing and breastfeeding outcomes (Alio et al., 2010; Banks et al., 2013; Martin et al., 2007; Sweet & Darbyshire, 2009). However, studies have also shown the negative effect of lower household incomes on PI (Alio et al., 2010; Carlson & Magnuson, 2011; Martin et al., 2007; Redshaw & Henderson, 2013). Our research in exploring the experiences of fathers with the WIC programme could be interpreted to reinforce prior findings as only two fathers met the definition of full participation. Even with the small sample size, this implies that WIC may be a missed opportunity to incorporate fathers in maternal and child support services.

The use of the Social Ecological Model illustrates how each level of the model, along with their interactions, frame the barriers to PI and also provides a roadmap of how these barriers might some be overcome. However, each barrier requires additional insight and understanding from fathers, mothers, community members, the WIC programme, and even at the level of policy-makers.

### Individual level

Encompassing Pride of Masculinity, this level illustrated threats related to men’s sense of masculinity. However, these fears were only expressed by the female participants highlighting a fear that even discussions about masculinity maybe taboo among men. It may also be indicative of the ongoing effect of toxic masculinity that seems to be pervasive in communities of colour (Bueno, 2018). This barrier may also be illustrative of the conflicting roles of nurturer and provider that seems to be more pervasive in these same communities (Haynes, 2000). Men continue to be torn between these roles especially as society questions their masculinity when they are characterized as unable to provide, such as when a family applies for assistance from a safety net programme like WIC. If men must continue to choose between the two roles, it seems that both masculinity and society favour the provider role making it a priority over WIC attendance and even enrolment.

### Interpersonal level

Characterized by Fears of Coercion and the Unacknowledged Male Paternal Role, this level highlights the conflict between the roles of provider and nurturer as expressed in the personal level. On one hand, fathers and mothers shared that men did not want to be coerced into WIC attendance, a role in obvious conflict with being the provider. Yet, families also expressed a fear that fathers were being excluded from the programme in support of their roles as nurturers. These two barriers illustrate the ongoing conflict between these two roles and acknowledges that before society can address its inability to balance the two roles, it must first happen at the interpersonal level.

### Community level

The barrier regarding fears of being excluded expand upon the same arguments around provider versus nurturer as highlighted at both the individual and interpersonal levels. However, unique to this level was the barrier of fear tied to feelings of distrust of government entities and authority figures. The WIC programme, like many US safety net programmes, has an income threshold based on the number of household members. While it was not shared by any of the participants, it is well known among WIC staff that fathers do not want their income included when the determination for benefits is made, as many fear it would result in a disqualification. Rather than risk the father being questioned about income, he either remains in the lobby or stays away completely. So, in many ways, that fear of privacy is justified, especially in cases where the family truly does not qualify for support.

Income thresholds, however, are not the only deterrent to participation nor the only reason for men to be fearful of programmes like WIC. The literature supports that there may be bias against men and their deserving of or need for support from social services agencies (Baum, 2016; Cameron et al., 2012). While fathers cited concerns over lack of privacy when sharing private information, it may not make a difference if they are already being judged for simply being at the providers’ desk.

### Organizational level

While components of the WIC programme, including programme interactions and office environment, were characterized as a barrier to PI, the concerns may be more of a perception issue rather than deliberate action on the part of WIC to discourage it. For example, participants cited a concern over sharing personal and financial information with some expressing fears about being overheard or having their information known by others. While some WIC locations utilize cubicles, rather than traditional offices in meeting with clients, effort is made to keep conversations discreet. WIC staff are also required to maintain the privacy and confidentiality of all data collected. In addition, in accordance with the Health Insurance Portability and Accountability Act (HIPAA), WIC had recently taken client privacy further by instituting a number system for calling clients to a particular station within the office rather than calling out a client’s name. However, as discussed in the Community Level, the barriers regarding interaction with staff may support prior research regarding bias against men documented in social agencies (Baum, 2016; Cameron et al., 2012). Results do indicate that there are opportunities for WIC to address participants concerns by reviewing operational and administrative processes, including the office environment. If perceptions are discouraging PI, WIC may consider reviewing those perceptions, to look for opportunities to foster inclusivity among all family members.

Participants, especially fathers, also shared specific concerns regarding their interactions with WIC staff including feelings of not being welcomed, being ignored, and WIC being unequal in their treatment of men and women. While some of these feelings could be addressed in creating a more welcoming environment, WIC’s treatment of men may also be influenced by the mother’s role of gatekeeper (Cannon et al., 2008; McBride et al., 2005). Specifically, WIC staff may be looking for the mother to give her permission in allowing her partner to participate in a programme some deem as “mother-safe”. If in the role of gatekeeper, the mother does not give that permission, no matter how involved that father is in his family’s life, he will not get past the lobby. Of course, none of the female participants shared that they assumed that role nor that their permission was needed for the father’s involvement.

Finally, both mothers and fathers shared concerns over the office environment, sharing how it was lacking in its depiction of fathers or of the family unit. There was also a lack of fathers in WIC marketing and educational materials with only one brochure focused on the role and support of fathers. Messages likes these, whether intentional or not, may also have contributed to feelings of exclusion as illustrated at the community level shared by fathers and mothers. Coupled with the WIC name along with billboards, photos, and brochures that elevated and celebrated motherhood, a typical WIC office had nothing a father could identify with nor picture himself being a part of.

### Policy

While semantics would dictate the word is not the thing, feelings around WIC’s less than inclusive name was brought up by several of the study participants. While benefits of the programme do not extend to men, the name alone could prevent fathers from enrolling qualifying children that are in their care. As such, this denies access to nutrition education to these families and limits their purchasing power in buying nutritious foods in support of healthy growth and development.

While the minimal role of fathers in the WIC programme may not be indicative of the man’s participation during pregnancy whether measured as social, emotional, physical or financial support; it may be indicative of the quality especially if he is not knowledgeable in prenatal health or nutritional needs. However, if lack of paternal involvement in WIC is associated with a lack of paternal involvement in pregnancy regardless of its measure, the argument could be made that there is a further association between rates of preterm and low birthweight births, as well as rates of breastfeeding (Centers for Disease Control and Prevention, 2013; Khanuja, 2017) among WIC-recipients as compared to the general population. This is further highlighted by measures of paternal involvement, which are lower among families of colour (Redshaw & Henderson, 2013; Surkan et al., 2017), as well as rates of preterm and low birthweight births among WIC recipients that exceed national rates, especially among African American families (Kline et al., 2020).

If WIC was to change perceptions of the programme by enacting real change such as creating gender inclusive environments, expanding staff training to include gender competency, marketing the programme to families, and finding ways to work with families where the mother and father are estranged, is it not possible that the additional participation of fathers could result in improved in birthing results, as well as the longer-term of improved family dynamics and fathers that remain active in their children’s lives well after delivery.

### Study strengths and limitations

The main strength of this study is that it fills a gap in research regarding the barriers to paternal involvement and household participation in the WIC programme, particularly among low-income families. In addition, it afforded the researchers to build strong community partnerships with participating mothers, fathers, and WIC employees. This study also builds on prior research on the role of men in pregnancy outcomes.

Study limitations included the small sample population, which may not have been representative of the population. Among participants, only three were Caucasian, while seven had at least some college. Additionally, nine participants had household incomes higher than 40,000 USD/year as compared to the 2018 federal poverty limit of 25,100 USD for a family of four (HHS, 2018)). These higher incomes may actually have been a deterrent to PI, as WIC staff shared that men did not attend WIC, as they did not want their incomes to disqualify a family for benefits. It should also be noted that while study participants were recruited as couples, there was no requirement that they resided together.

### Future research

Future research should ensure sampled populations are more representative of WIC enrolments, possibly using other community resources to recruit. As this study was a pilot, we may need to review our data collection procedure and methods to capture additional information about male experiences with WIC pertaining to perceptions of masculinity and the role of fathers. We may also need to include living situation to capture the domicile/residential arrangements between mother and father.

Our findings indicate a need to increase PI by considering multi-component interventions aligned with the SEM. Future research could investigate the individual barriers as well as analyse their intersectionality. This includes barriers attributed to societal norms, as the role of men and fatherhood are defined differently from racial, ethnic, and religious perspectives. A community-based participatory research (CBPR) approach may be employed to help families get more involved in the research planning and implementation phase by advocating for researchers to focus on factors and interventions which might positively influence PI. Future directions for this research include conducting a process analysis of the WIC programme, including an assessment of gender competency concerning its policies, administration and operations throughout the entire period of enrolment.

## Supplementary Material

Supplemental Material
