# Supplementary material for: “When a Father feels Excluded”: A Qualitative Study Exploring the Role of Fathers in the Women, Infants, and Children (WIC) Supplemental Nutrition Program
Source: Int J Qual Stud Health Well-being. 2021 Jun 22;16(1):1932026. doi: 10.1080/17482631.2021.1932026 (PMC8221125; doi:10.1080/17482631.2021.1932026)
Supplement: Supplemental Material [file ZQHW_A_1932026_SM9707.docx]

**Supplementary Table. Summary of sub-themes based on the Social-Ecological Model**

| SEM Level | Sub-Theme | Description | Related Quotes |
| --- | --- | --- | --- |
| Individual | Pride of masculinity | Feelings of pride related to gender. Any threat to that pride or self-esteem. | *"It's a pride thing for men…don’t like to discuss things in front of others…privacy very important" (African American mother of 3, age 31)* |
|  |  |  | *"A lot of males think WIC is welfare…some people think welfare is bad" (African American mother of 3, age 33)* |
| Interpersonal | Unacknowledged male paternal role | Feelings of not having role of contribution acknowledged | *"Men feel like they don’t get credit as a parent…domino effect where the father feel touched by the program lifts his entire family" (African American mother of 3, age 31)* |
|  |  |  | *"When you say women, infants, and children, I think they're missing the…the key factor which is the mom and dad" (African American mother of 3, age 31)* |
|  |  |  | *"You want to see happy families and whole families and I think this gives people a chance to…push and promote that" (African American mother of 3, age 31)* |
|  | Fears of coercion | Paternal fears of being forced attend; lack of personal choice | *"WIC should not play a role in increasing father involvement, it's being forced…it must be voluntary" (African American father of 3, age 35)* |
|  |  |  | *"If you say WIC should increase my role, that's kind of being forced" (African American father of 3, age 35)* |
|  |  |  | *"WIC should be involved with increasing father participation as long as it's not invasive or forced" (African American father of 5, age 37)* |
| Community | Fear | Fear of authority or government | *"I think a lot of minorities and low-income families are afraid to ask at the hospital because they're scared a social worker or case worker will be invited in" (African American mother of 3, age 31)* |
|  | Feelings of exclusion | Expressed or subverted feelings of being excluded | *"A lot of fathers…are fathers before they're even men, you know? And they, we grow up without fathers. We don’t really know how to care for a kid, you don’t know how to be a dad, you never had one" (African American mother of 2, age 24)* |
|  |  |  | *"When a father feels excluded, he wants be less involved…if they feel these people want me around, it helps them better support the woman" (African American father of 3, age 38)* |
| Organizational | Program interactions | Issues related to interactions between fathers and the WIC program, including interactions with WIC personnel | *"They don’t make the male uncomfortable, but they're expecting a female" (African American father of 3, age 38)* |
|  |  |  | *"Fathers are playing an important role and that's sometimes an ignored role" (Caucasian father of 2, age 38)* |
|  |  |  | *"Men not made to feel special…not welcoming to men" (African American father of 3, age 35)* |
|  |  |  | *"Adopt a more gender-neutral framework for parenting" (Caucasian father of 2, age 38)* |
|  |  |  | *"WIC puts men through more then they do moms" (African American father of 3, age 35)* |
|  |  |  | *"Fathers would benefit a lot from wholesale changes to WIC's approach…if WIC was to adopt some of these more holistic um, programs" (Caucasian father of 2, age 38)* |
|  |  |  | *"WIC could be…unintentionally a hindrance to families...if they're not reflecting it" (Caucasian father of 2, age 38)* |
|  |  |  | *"The program hadn't really accounted for dads" (Caucasian father of 2, age 38)* |
|  |  |  | *"They just have the mom's name on the card and I think if they involve the male, they need to have the guy's name on the card" (African American mother of 2, age 32)* |
|  | Office environment | Anything related to the look or feel of the office including furniture, graphics, marketing material or even the vibe | *"It's not welcoming to both participants whether its male or female" (African American father of 3, age 38)* |
|  |  |  | *"I find it unwelcoming as a person…like it's not a warm environment…it feels very cold" (Caucasian mother of 2, age 37)* |
|  |  |  | *"I felt like not, not bad about it, but the program hadn't really accounted for dads" (Caucasian father of 2, age 38)* |
|  |  |  | *"There were like literally no men at all in the like, whole area" (Caucasian mother of 2, age 37)* |
|  | Hours of operation | Issues related to WIC’s hours of operation | *"Her appointments are when I'm working and I'm not going to miss work to go to a WIC appointment" (African- American father of 5, age 37)* |
|  |  |  | *"Some people work and can't make it" (African American father of 2, age 26)* |
|  |  |  | *"They haven't been open on, at times that were convenient for me" (African American father of 3, age 35)* |
| Policy | Program Name | Anything related to the name of the program itself | *"Change the name of WIC to better reflect male inclusion…just the name can make men feel uncomfortable" (African American father of 3, age 38)* |
|  |  |  | *"When you say women, infants, and children, I think they're missing the…the key factor which is the mom and dad" (African American mother of 3, age 31)* |
